# Modeling COVID-19 infection dynamics and mitigation strategies for in-person K-6 instruction

**DOI:** 10.3389/fpubh.2023.856940

**Published:** 2023-02-07

**Authors:** Douglas E. Morrison, Roch Nianogo, Vladimir Manuel, Onyebuchi A. Arah, Nathaniel Anderson, Tony Kuo, Moira Inkelas

**Affiliations:** ^1^Department of Epidemiology, Fielding School of Public Health, University of California, Los Angeles, Los Angeles, CA, United States; ^2^Department of Biostatistics, Fielding School of Public Health, University of California, Los Angeles, Los Angeles, CA, United States; ^3^Department of Public Health Sciences, School of Medicine, University of California, Davis, Davis, CA, United States; ^4^Clinical and Translational Science Institute, University of California, Los Angeles, Los Angeles, CA, United States; ^5^Department of Family Medicine, David Geffen School of Medicine, University of California, Los Angeles, Los Angeles, CA, United States; ^6^Department of Statistics, College of Letters and Science, University of California, Los Angeles, Los Angeles, CA, United States; ^7^Department of Health Policy and Management, Fielding School of Public Health, University of California, Los Angeles, Los Angeles, CA, United States

**Keywords:** COVID-19, school district, agent-based model, secondary transmission, K-6

## Abstract

**Background:**

U.S. school closures due to the coronavirus disease 2019 (COVID-19) pandemic led to extended periods of remote learning and social and economic impact on families. Uncertainty about virus dynamics made it difficult for school districts to develop mitigation plans that all stakeholders consider to be safe.

**Methods:**

We developed an agent-based model of infection dynamics and preventive mitigation designed as a conceptual tool to give school districts basic insights into their options, and to provide optimal flexibility and computational ease as COVID-19 science rapidly evolved early in the pandemic. Elements included distancing, health behaviors, surveillance and symptomatic testing, daily symptom and exposure screening, quarantine policies, and vaccination. Model elements were designed to be updated as the pandemic and scientific knowledge evolve. An online interface enables school districts and their implementation partners to explore the effects of interventions on outcomes of interest to states and localities, under a variety of plausible epidemiological and policy assumptions.

**Results:**

The model shows infection dynamics that school districts should consider. For example, under default assumptions, secondary infection rates and school attendance are substantially affected by surveillance testing protocols, vaccination rates, class sizes, and effectiveness of safety education.

**Conclusions:**

Our model helps policymakers consider how mitigation options and the dynamics of school infection risks affect outcomes of interest. The model was designed in a period of considerable uncertainty and rapidly evolving science. It had practical use early in the pandemic to surface dynamics for school districts and to enable manipulation of parameters as well as rapid update in response to changes in epidemiological conditions and scientific information about COVID-19 transmission dynamics, testing and vaccination resources, and reliability of mitigation strategies.

## 1. Background

School closures due to the coronavirus disease 2019 (COVID-19) pandemic led to extended periods of remote learning, with potential harm for children's educational progress, psychosocial development, and mental and physical health (1–4). School closures also affect families, workplaces, and workforce participation (5). Since COVID-19 burden is often greater in socioeconomically disadvantaged communities, in-person instruction early in the pandemic was unavailable or inconsistent for the students who may need it the most, thereby increasing educational disparities for children in lower income families, communities of color, and households that include essential workers, contain multiple generations, experience crowded housing, and with members who have chronic conditions that put them at risk for severe COVID-19 (6, 7). Therefore, school districts need to find ways to bring all students back safely.

Recent studies suggest that secondary infection risk in schools is low when basic precautions are followed (8–10). Yet uncertainty surrounding the infectiousness of evolving viral variants and the management of pre- and asymptomatic populations slowed and disrupted school re-openings in the U.S. and has affected school attendance once re-opened. Maintaining in-person instruction, and consistent school attendance, during the pandemic means reducing infection hazard for susceptible children and adults who congregate daily for extended periods of time.

School districts sought to work with their public health authorities to understand and act upon risk in a dynamic environment, using the policy levers available to them. There is a need for practical models that inform planning for safer in-person instruction in K-6 settings. A practical model surfaces possible infection dynamics and is flexible in parameters and their values and is designed computationally to provide rapid results. Transparent flexible models could facilitate deeper understanding about levers of influence among local authorities tasked with responding to the pandemic on a day-to-day basis (11).

We designed a stochastic, agent-based model of resumed in-person instruction that includes representations of a variety of intervention levers, including screening for symptoms and exposures, biological testing, education to reduce transmission risks from socializing without distancing or masking, and vaccination. We developed this model while collaborating as a university science partner with a large urban school district to consider what would be necessary for safer resumption of this much needed face-to-face learning. Because the science of COVID-19 was evolving rapidly, we sought to create a model with parameters that end users could easily adjust as more information emerged regarding transmission dynamics and the impact of mitigation strategies. The model was flexible to accommodate different school structures and local environments.

This model was designed for adaptation; we implemented the model in the R statistical computing environment (v4.1.0) (12) and provide the source code at https://github.com/UCLA-PHP/school.epi.abm. We used the model to assess key outcomes of interest to school district stakeholders, and we provided an online user interface to the model as a practical tool to empower school districts and their implementation partners to explore how various combinations and variations of strategy components affect health and learning outcomes within different underlying epidemiological conditions that could arise in the real world. This user interface can be accessed at https://agent-based-models.shinyapps.io/RegionalCOVIDSchoolSimulation/. The purpose of this paper is to showcase the capabilities of this simulation model, not to make specific predictions for a particular school district or epidemiological scenario. We hope that this model will be helpful for policymakers both in future stages of the COVID-19 pandemic and for future epidemics.

## 2. Methods

### 2.1. Model design and scope

Here we describe the model in general terms; a detailed description of the implemented model is provided as Supplementary material.

#### 2.1.1. Agents

The model contains two types of simulated individuals (“agents”): students and their associated household adults (two per student). We chose to include these agent types as they form the largest proportion of the school community. The model could be extended to include teachers and other school personnel as additional agents who interact with students and with one other. Each student is assigned to a particular school and classroom and has several “close classmates” within their class; close classmates have higher risks of transmission than other classmates.

#### 2.1.2. Sources of infection

Infections in the model come from three sources: infectious classmates at school, infectious family members at home, and exogenous exposures outside of school and home.

On each day of the simulation, each currently infectious student who is currently in school has a chance to infect each other student in their class who is not yet infected. The risk of infection for a given student is $1\;-\;{\left(1\;-\;p_{C}\right)}^{C}{\left(1-p_{D}\right)}^{D}$, where *C* is the number of infectious close classmates currently in attendance, *p*_*C*_ is the parameter for the risk of transmission to close classmates per infectious student (the “effective contact risk” for close classmates), *D* is the number of infectious contacts (including both close and distant classmates) currently in attendance, and *p*_*D*_ is the parameter of risk of transmission to distant classmates per infectious student. For example: if on a given day, a particular student has 2 infectious close classmates and 3 infectious distant contacts currently at school with them, then if *p*_*C*_ = 0.01 and *p*_*D*_ = 0.005, that student has a [1−(1 − 0.01)^2^(1 − 0.005)^2+3^] × 100%≈4.4% chance of being infected in school on that day.

Infectious students have a chance to infect their household adults, and infectious household adults have a chance to infect their students and a chance to infect the other household adult (if not already infected). For easier interfacing with the available literature, the daily transmission risks are specified indirectly. The user interface provides parameters for the risk of transmission per infection. The risk per day is calculated based on this parameter and the duration-of-infectiousness parameters (“infection time-course”), as:


$$\begin{array}{l} risk\;per\;infectious\;day\;=1-{\left(1-risk\;per\;infection\right)}^{1/\#\;days\;infectious}. \end{array}$$

Finally, each student has a daily exogenous risk of infection outside of school and home, which depends on whether they have received COVID-19 safety education (as described in the Interventions section below). Each household adult also has a risk of exogenous infection.

#### 2.1.3. Infection progression

Agents follow a susceptible-infected-recovered (SIR) framework in which they are initially either susceptible to infection, infected, or vaccinated or recovered (i.e. immune) (13). Susceptible individuals can become infected over the course of the simulation. Infected individuals then progress through a series of infection states. They first enter a latent period during which they are not yet infectious or symptomatic. Next, they become infectious but presymptomatic. Infectious presymptomatic individuals can become symptomatic or remain asymptomatic. Eventually, infected individuals recover and become immune.

#### 2.1.4. Interventions

The model includes representations of several possible program components for resuming and maintaining in-person instruction. Not all these components may be implemented in some school districts, so the model has options for some of these components to be deactivated. For example, surveillance testing of non-symptomatic individuals can be eliminated by setting the “testing fraction” for surveillance testing to 0%. Thus, model users can modify parameters to exclude or alter some of the mitigation strategies to represent scenarios relevant to their environment.

One component is a daily symptom/exposure screening system through which students self-report if they have COVID-19 exposures or symptoms. This daily health screening may reduce the rates of infectious individuals coming onto campus; individuals reporting symptoms or suspected COVID-19 exposures could be diverted into quarantine protocols or receive other triage and follow-up. Such screenings have been implemented in workplaces, universities, and K-12 systems (14).

Another possible component of school-based mitigation strategies is outreach education of school community members. In such a program, school representatives might make phone calls to students' family members to provide information and guidance about safe behaviors that reduce their exposure to COVID-19 (e.g., social distancing, mask usage, and vaccination). In addition to influencing behavior, such engagement with students and their families may change the likelihood that individuals accurately report potential exposures and symptoms on the daily screen. The model includes a representation of this type of educational outreach.

Testing for COVID-19 infection is another possible component. Tests can be performed in response to reported COVID-19 symptoms or known exposure. Periodic surveillance testing can also be performed universally or in a random sample of non-symptomatic individuals to identify presymptomatic and asymptomatic cases. For the 2021–2022 school year, our partner district implemented weekly surveillance testing of 100% of their students. Both responsive and surveillance testing are represented in the model. The model can represent tests with different accuracy characteristics (specificity, sensitivity as a function of elapsed time since infection). In the analyses below, we assumed accuracy characteristics similar to PCR testing, but these parameters and testing frequency could be changed to represent antigen testing (15).

Other policies that can influence infection risks at school include defining and maintaining small groups in close proximity (e.g., classrooms, lunch groups) as well as using masks, physical space dividers, and other forms of physical barriers (16).

### 2.2. Model outcomes

We report the following model outcomes after 2 months of simulated full-time in-person school:

1. The cumulative percentage of enrolled students infected with COVID-19 since baseline.
2. The cumulative percentage of enrolled students infected with COVID-19 while at school.
3. The cumulative number of school days missed per student.
4. The percentage of schools with no in-school transmissions.
5. The percentage of schools with no detected infection clusters.

Other measured outcomes, such as infection rates among household adults, are not reported here in the interest of brevity but are provided in the online interface.

For each experimental scenario considered below, we simulated 10,000 schools in a single run of the model using the corresponding set of input parameter values. We calculated the five outcomes listed above for each school, and then combined results across the simulated schools to estimate outcome distribution summary statistics. For the student-level outcomes (#1-3), we report the means across the 10,000 simulated schools, as well as the 2.5 and 97.5% percentiles of these outcomes as 95% prediction intervals. For the school-level outcomes (#4-5) we report the event rates as percentages, as well as 95% exact binomial confidence intervals (percentiles and prediction intervals are not applicable for these outcomes).

### 2.3. Validation

Validation tests confirmed that the agent-initializing function produced the specified initial rates of current infection, prior infection, and COVID safety education characteristics among students at baseline for the default input parameter values. In the absence of school data when the model was developed, it was not feasible to calibrate this model. Methodological constraints make it difficult to compare the model output to actual experience of school districts; for example, few districts have reliable estimates of COVID-19 positivity from well-designed surveillance. For this paper, we assigned default parameter values based on the existing literature where possible and considered likely values for variables with considerable uncertainty (details in Supplementary material). To use the model to inform planning, policymakers should choose parameter values that reflect current conditions in their schools.

### 2.4. Example experiments

To demonstrate how the model can be used to explore the effects of interventions, we tested the effects of changes in four parameters that could be affected by school policies: class size, frequency of surveillance testing, fraction of students tested in each surveillance sample (“sampling fraction”), and proportion of household adults vaccinated (Tables 1–4). We started with the default parameter values and varied these four parameters to determine how the outcomes changed in response. We also considered a set of four scenarios examining interactions between surveillance testing and community education (Table 5).

**Table 1:** Mean-average outcomes after 2 months of in-person instruction, by class size.

**Number of students per class**	% of students infected since baseline (cumulative)^*^	% of students infected from school (cumulative)^*^	# School days quarantined per student (cumulative)^*^	% of schools with no on-campus transmissions so far^**^	% of schools with no detected infection clusters so far^**^
10	4.48 (2.62, 6.43)	0.04 (0.00, 0.24)	1.21 (0.91, 1.53)	84.2% (83.5, 84.9)	99.3% (99.1, 99.5)
15 (default)	4.50 (2.62, 6.67)	0.05 (0.00, 0.24)	1.21 (0.91, 1.53)	80.2% (79.4, 81.0)	99.0% (98.7, 99.1)
20	4.51 (2.62, 6.67)	0.06 (0.00, 0.48)	1.21 (0.92, 1.56)	76.9% (76.1, 77.7)	98.0% (97.7, 98.3)
30	4.53 (2.62, 6.67)	0.09 (0.00, 0.48)	1.24 (0.92, 1.83)	69.3% (68.4, 70.3)	94.8% (94.3, 95.2)

* 2.5 and 97.5% percentiles (across the 10,000 simulated schools) are provided for student-level outcomes.

** 95% exact binomial confidence intervals are provided for school-level outcomes.

**Table 2:** Mean-average outcomes after 2 months of in-person instruction, by surveillance testing frequency and sampling fraction.

**Sampling fraction**	**Surveillance testing schedule**	% of students infected since baseline (cumulative)^*^	% of students infected from school (cumulative)^*^	# School days quarantined per student (cumulative)^*^	% of schools with no on-campus transmissions so far^**^	% of schools with no detected infection clusters so far^**^
0%	No surveillance testing	4.49 (2.62, 6.67)	0.06 (0.00, 0.48)	1.18 (0.89, 1.49)	79.2% (78.4, 80.0)	99.5% (99.4, 99.7)
25%	Once a week (M) (default)	4.50 (2.62, 6.67)	0.05 (0.00, 0.24)	1.21 (0.91, 1.53)	80.2% (79.4, 81.0)	99.0% (98.7, 99.1)
	Twice a week (M/Th)	4.50 (2.62, 6.67)	0.05 (0.00, 0.24)	1.23 (0.94, 1.58)	81.3% (80.6, 82.1)	97.9% (97.6, 98.1)
	3x a week (MWF)	4.48 (2.62, 6.67)	0.05 (0.00, 0.24)	1.25 (0.95, 1.61)	83.0% (82.3, 83.8)	96.8% (96.4, 97.1)
	Every weekday (M-F)	4.49 (2.62, 6.67)	0.04 (0.00, 0.24)	1.30 (0.99, 1.68)	84.2% (83.5, 84.9)	94.5% (94.1, 95.0)
100%	Once a week (M)	4.49 (2.62, 6.67)	0.04 (0.00, 0.24)	1.28 (0.97, 1.65)	84.7% (84.0, 85.4)	95.7% (95.3, 96.1)
	Twice a week (M/Th)	4.48 (2.62, 6.43)	0.04 (0.00, 0.24)	1.35 (1.02, 1.78)	86.2% (85.5, 86.9)	90.5% (89.9, 91.0)
	3x a week (MWF)	4.47 (2.62, 6.43)	0.03 (0.00, 0.24)	1.43 (1.09, 1.90)	87.7% (87.0, 88.3)	83.9% (83.2, 84.6)
	Every weekday (M-F)	4.48 (2.62, 6.67)	0.03 (0.00, 0.24)	1.59 (1.20, 2.14)	87.3% (86.7, 88.0)	70.2% (69.3, 71.1)

* 2.5 and 97.5% percentiles (across the 10,000 simulated schools) are provided for student-level outcomes.

** 95% exact binomial confidence intervals are provided for school-level outcomes.

**Table 3:** Additional mean-average outcomes after 2 months of in-person instruction, by surveillance testing frequency and sampling fraction.

**Sampling fraction**	**Surveillance testing schedule**	# School days quarantined and uninfectious per student (cumulative)^*^	# School days quarantined and infectious per student (cumulative)^*^	# School days on-campus and infectious per student (cumulative)^*^
0%	No surveillance testing	1.02 (0.78, 1.29)	0.15 (0.07, 0.25)	0.06 (0.01, 0.12)
25%	Once a week (M) (default)	1.05 (0.80, 1.33)	0.16 (0.07, 0.25)	0.05 (0.01, 0.11)
	Twice a week (M/Th)	1.07 (0.82, 1.37)	0.16 (0.07, 0.26)	0.05 (0.01, 0.10)
	3x a week (MWF)	1.09 (0.84, 1.40)	0.16 (0.07, 0.26)	0.05 (0.01, 0.10)
	Every weekday (M-F)	1.13 (0.86, 1.47)	0.16 (0.07, 0.27)	0.04 (0.01, 0.09)
100%	Once a week (M)	1.11 (0.85, 1.43)	0.17 (0.08, 0.27)	0.04 (0.01, 0.08)
	Twice a week (M/Th)	1.18 (0.89, 1.57)	0.17 (0.08, 0.27)	0.04 (0.01, 0.07)
	3x a week (MWF)	1.26 (0.95, 1.69)	0.17 (0.08, 0.28)	0.03 (0.01, 0.06)
	Every weekday (M-F)	1.41 (1.06, 1.93)	0.18 (0.09, 0.28)	0.03 (0.01, 0.06)

* 2.5 and 97.5% percentiles (across the 10,000 simulated schools) are provided for these student-level outcomes.

**Table 4:** Mean-average outcomes after 2 months of in-person instruction, by adult vaccination rate.

**Percentage of household adults vaccinated**	% of students infected since baseline (cumulative)^*^	% of students infected from school (cumulative)^*^	% of students infected by a household adult (cumulative)^*^	% of students not yet infected (cumulative)^*^	# School days quarantined per student (cumulative)^*^	% of schools with no on-campus transmissions so far^**^	% of schools with no detected infection clusters so far^**^
0% (default)	4.50 (2.62, 6.67)	0.05 (0.00, 0.24)	1.27 (0.24, 2.38)	91.5 (94.1, 88.6)	1.21 (0.91, 1.53)	80.2% (79.4, 81.0)	99.0% (98.7, 99.1)
25%	4.15 (2.38, 6.19)	0.06 (0.00, 0.48)	0.92 (0.24, 1.90)	91.9 (94.3, 89.3)	1.06 (0.79, 1.35)	79.5% (78.7, 80.3)	98.9% (98.6, 99.1)
50%	3.86 (2.14, 5.95)	0.05 (0.00, 0.48)	0.62 (0.00, 1.43)	92.1 (94.5, 89.5)	0.92 (0.69, 1.19)	80.0% (79.3, 80.8)	98.7% (98.5, 98.9)
75%	3.58 (1.91, 5.48)	0.06 (0.00, 0.48)	0.34 (0.00, 0.95)	92.4 (95.0, 89.8)	0.79 (0.59, 1.03)	78.8% (78.0, 79.6)	99.0% (98.7, 99.1)

* 2.5 and 97.5% percentiles (across the 10,000 simulated schools) are provided for student-level outcomes.

** 95% exact binomial confidence intervals are provided for school-level outcomes.

**Table 5:** Scenarios assessing the effects and interactions of surveillance testing and educational outreach to families.

**Scenario Description**	**Surveillance testing frequency**	**Probability of outreach receptiveness**	**Improvements in attestation accuracy and exogenous risk from COVID safety education**	% of students infected since baseline (cumulative)^*^	% of students infected from school (cumulative)^*^	# School days quarantined per student (cumulative)^*^	% of schools with no on-campus transmissions so far^**^	% of schools with no detected infection clusters so far^**^
Default parameter values	25% every Monday	50%	10%	4.50 (2.62, 6.67)	0.05 (0.00, 0.24)	1.21 (0.91, 1.53)	80.2% (79.4, 81.0)	99.0% (98.7, 99.1)
No testing or education	None	0%	50%	4.33 (2.62, 6.43)	0.05 (0.00, 0.48)	1.14 (0.85, 1.45)	80.4% (79.6, 81.2)	99.5% (99.3, 99.6)
Education only	None	100%	50%	4.10 (2.38, 6.19)	0.04 (0.00, 0.24)	1.08 (0.80, 1.38)	83.8% (83.1, 84.5)	99.7% (99.5, 99.8)
Testing only	100% every Monday	0%	50%	4.33 (2.38, 6.43)	0.04 (0.00, 0.24)	1.24 (0.94, 1.60)	84.9% (84.2, 85.6)	95.7% (95.3, 96.1)
Testing + education	100% every Monday	100%	50%	4.07 (2.38, 6.19)	0.03 (0.00, 0.24)	1.17 (0.89, 1.52)	88.3% (87.6, 88.9)	96.4% (96.0, 96.8)

* 2.5 and 97.5% percentiles (across the 10,000 simulated schools) are provided for student-level outcomes.

** 95% exact binomial confidence intervals are provided for school-level outcomes.

### 2.5. Sensitivity analyses

In sensitivity analyses, we tested the effects of additional parameters: number of non-socially distanced classmates per student, test sensitivity and specificity, transmission risk from infectious students to non-socially-distanced classmates, exogenous infection risk, symptom/exposure reporting sensitivity and specificity prior to COVID-19 safety education outreach, receptiveness to COVID-19 safety education outreach, and effects of COVID safety education on accuracy of symptom/exposure reporting, and exogenous risk (Supplementary material). We created tornado plots as a simple, interpretable display; these plots assess the relative importance of these variables with respect to each outcome (17).

## 3. Results

In 10,000 schools simulated for 2 months of in-person instruction using the default parameter values except for class size, smaller class sizes resulted in fewer students infected in-school, more schools remaining transmission-free, and fewer school days missed (Table 1). With classes of 30 students each, an average of 4.53% of students became infected, 0.09% were infected in-school, 1.24 school days were missed per student, and 69.3% of schools remained transmission-free. With 10 students per class, these outcomes improved to 4.48% infected overall, 0.04% infected in-school, 1.21 school days missed per student, and 84.2% of schools remaining infection-free.

More surveillance testing resulted in lower transmission rates, but more school days missed (Table 2). With no surveillance testing, 4.49% of students became infected after baseline, 0.06% were infected at school, 1.18 school days were missed per student, and 79.2% of schools had no on-campus transmissions. Weekly surveillance testing with a randomly selected 25% of the student body tested in each week did not change these outcomes substantially. Daily testing of 25% of the student body produced small improvements in infection rates: 4.49% of students became infected overall, 0.04% were infected in school, and 84.2% of schools remained transmission-free; however, average school days missed increased to 1.30 days per student. Finally, daily testing of all students produced larger improvements in secondary infection rates: 4.48% of students were infected overall, 0.03% in school, and 87.3% of schools stayed transmission-free; however, school days missed rose to 1.59 days per student. The percentage of schools with no detected clusters had an opposite trend to the percentage of schools with no actual transmissions: with more surveillance testing of asymptomatic students, more schools had clusters detected.

The observed tradeoff between transmission and attendance occurred because increased testing increased the numbers of true positive cases, which are correctly isolated, but also the numbers of false positive cases, which are unnecessarily isolated: with no testing, students spent an average of 0.06 school days on-campus and infectious, vs. 0.03 school days with daily 100% testing; however, with 100% daily testing, students also averaged 1.41 school days quarantined and uninfectious, vs. 1.02 days with no surveillance testing (Table 3).

Higher levels of vaccination among household adults resulted in fewer infections overall and fewer school days missed, but no improvements in on-campus infections (Table 4). With no vaccinations, 4.50% of students became infected since baseline, 0.05% were infected while at school, 1.27% of students were infected by a household adult, 91.5% of students remained uninfected, 1.21 days were missed, and 80.2% of schools had no on-campus transmissions. With 75% of the adults vaccinated, only 3.58% of students became infected since baseline and 0.79 school days were missed per student; 0.06% of students were infected on campus, 0.34% were infected by a household adult, 92.4% remained uninfected, and 78.8% of schools had no on-campus transmissions. The decrease in the proportion of schools with no on-campus transmissions between the 0 and 75% adult vaccination scenarios was small (1.4 percentage points) but statistically significant (continuity-corrected chi-square test *p* = 0.02); as fewer students were being infected at home (0.34 vs. 1.27%), more remained uninfected and hence vulnerable to infection at school (92.4 vs. 91.5%).

In the scenarios examining interactions between surveillance testing and school community education, the “Education only,” “Testing only,” and “Testing + Education” scenarios all resulted in lower infection rates than the “No testing or education” scenario (Table 5). “Testing only” had better in-school infection rates than “Education only” but worse overall infection rates and average attendance rates. “Testing + Education” produced better infection rates than either strategy alone and a better average attendance rate than “Testing alone.”

### 3.1. Sensitivity analyses

Detailed sensitivity analysis results are provided in the Supplementary material. Starting from our default assumptions, in-school infections were most affected by changes in risk per infectious classmate, exogenous infection risk, exposure and symptoms screening sensitivity and specificity, likelihood of symptoms if infected, class size, number of non-distanced classmates in school, and biological test specificity, while attendance rates were most affected by changes in symptoms/exposure screening specificity, exogenous infection risk, biological test specificity, and vaccination rate.

## 4. Discussion

Our model provides results in terms of health as well as attendance. Key findings are that (1) as expected, smaller class size resulted in less school transmission as well as fewer missed school days; (2) frequent testing leads to reduced transmission but increased school days missed due to positive students as well as students with false positive tests being in extended home isolation; and (3) vaccination of household members reduces the number of school days missed per student and total students becoming infected but not school transmission. Comparing different combinations of mitigation strategies, with varying assumptions about the accuracy of parent-reported symptoms and exposures, produced results in school infection and attendance that were not additive, and show stakeholders how implementation integrity in combination with elected mitigation strategies could affect outcomes of interest.

Using parameters that reflect our best knowledge about COVID-19, the simulations show that no single program element or condition ensures safety and that some combinations have trade-offs between school infection and attendance. Our model reflects recent evidence that even without COVID-19 testing, on-campus infection control can reduce on-campus transmission, and high community prevalence does not necessarily lead to significant secondary infection if mitigation measures such as masking are implemented effectively (10), especially if vaccination rates are high.

Notably, the model illustrates the value of school districts measuring not just adoption of policy but the implementation quality of their mitigation strategies. For example, given the presymptomatic and asymptomatic features of COVID-19, particularly in children, the simulations help stakeholders appreciate the impact of accurate reporting of symptoms and exposures. School districts can see the potential impact of accurate parent and student reporting and therefore the potential need for effective design of the screening questions as well as ongoing education to improve the accuracy of reporting.

Public health credibility has been vital in the COVID-19 pandemic, and stakeholders need models that reflect the latest science so that district decisions are trusted. Our model is designed to be practical, transparent, and adaptable as mechanisms for transmission and mitigation and their interdependencies become known.

The model's structure and dynamics are not limited to COVID-19. With appropriate adjustment of the parameter values representing transmission risks and infection characteristics, this model could be used to represent any infectious disease and adapted for other congregate settings, such as residential facilities.

### 4.1. Limitations

This model was developed to surface dynamics that give stakeholders insights about mitigation strategies as the pandemic evolved. The model is not intended in its current form to make specific predictions or justify specific actions. Validation of the model's parameters and predictions with real data could increase its utility for accurately predicting policy outcomes. Notably, it is not possible to fully validate the model; most studies of COVID-19 prevalence and transmission in U.S. schools are limited by lack of systematic testing, incomplete contact tracing, and details about mitigation procedures as well as adherence to them and their timing (10, 18–20). Demonstrating the model in its current state provides a framework with outcomes that can improve how modelers provide and interpret results for school district stakeholders and provides a basis for future extensions.

In the simulation scenarios presented in this paper, we found that increasing vaccination rates for household adults resulted in improved student attendance rates (Table 4), but the size of this effect in practice will depend on the specific epidemiological, demographic, and policy factors that a particular school is currently experiencing. This model represents this context with a large number of modifiable input parameters, but there are inevitably additional dynamics which are not included in the model.

For example, characteristics of households of school community members that influence their exposure to COVID-19 include recurrent proximity to other household members (number in the household, and overcrowding), intermittent proximity to other individuals who do not live in the household (such as extended family/friends), density of neighborhood housing density (proxy for proximity), and ongoing potential workplace exposures such as essential or industrial workers in the household. Household behaviors include close physical contact, multiple caregivers of a child, and uses of facial coverings and other safety practices. Household health risks such as presence of individuals with chronic conditions, and/or older age, influences impact of household morbidity from any school-transmitted COVID-19 infection. None of these characteristics are implemented in the current model, for succinctness and due to limited resources for further extending the model, but they may play an important role in local transmission dynamics, particularly since these risk factors often co-occur with one another.

We also made a simplifying assumption that vaccination and recovery from infection each independently confer complete, long-term immunity from future infection. At the time when this model was being developed (Q4 2020), this assumption was plausible. Since that time, both scientific knowledge and the COVID-19 virus itself have evolved substantially, and the immunities conferred by vaccination or prior exposure are now understood to be incomplete, diminishing over time, and dependent on the specific type of vaccine received and on the variants of the COVID-19 virus that an individual has previously recovered from, compared to the one they are currently being exposed to. Immunity could be modeled with more nuance in future extensions of this model, as discussed below.

### 4.2. Future directions

There are three main avenues for further development of this work. First, the model could be extended, adding other agents such as teachers and other school personnel; incorporating more complicated social networks including sibling connections and asymmetric exposures; other interactions such as shared transportation (school buses and carpools) and after-school sports; compliance (reliability) in mitigation such as handwashing and mask-wearing; more nuanced dynamics for test sensitivity, for example having test sensitivity depend explicitly on symptomatic status rather than only days since infection; and imperfect and time-dependent immunity to infection after vaccination or recovery from prior infection. Second, the interactions between different input parameters could be explored, by simultaneously varying multiple parameters instead of only one at a time as we have primarily done in this paper. This is a more realistic use of the model and how we envision public health authorities and school systems making use of it. Readers are encouraged to access the model *via* the user interface at https://agent-based-models.shinyapps.io/RegionalCOVIDSchoolSimulation/ to explore other combinations of input values. Third, the user interface could be augmented by adding side-by-side comparisons of the outcomes for different combinations of input parameter values, narrative descriptions of individual runs of the simulation, and additional outcome time series.

## 5. Conclusions

Models enable stakeholders and researchers to consider infection dynamics and potential mitigation strategies in combination. Public health authorities and school systems can use insights from these models to establish operational needs for safer in-person instruction, such as accurate daily health checks and ongoing timely data on the reliability of mitigation strategies. Models can facilitate an iterative process by which understanding of the system is further deepened, which can in turn be used to reassure communities that schools can deliver in-person instruction without triggering large outbreaks. With future calibration, the model can ultimately have value for prediction, especially as the pandemic eventually becomes endemic, and new transmission and disease control scenarios arise (21, 22).

Given the availability of highly effective vaccines and the amount of community infection with COVID-19, the predictions of this model for a school district would be limited. However, the model continues to be useful in demonstrating key inputs to viral dynamics. It is also an example of a model that includes key inputs and that allows real-time change by users of key parameters and assumptions. We have published the source code for the model on GitHub (https://github.com/UCLA-PHP/school.epi.abm) so that other researchers can use and extend the model for districts in any location.

It is important for decision-making models in COVID-19 to be flexible given the rapid evolution of knowledge about how the virus operates, the rapid transmission dynamics of a disease that spreads through a population exponentially, and the rapidly changing landscape of testing features, costs, and operational burdens as what is being seen in the most recent wave of the Omicron variant infection (23, 24).

Public health authorities and school districts can make more meaningful choices about the welfare of K-6 students, their teachers, and their families if these decisions about in-person instruction are based on information from models that incorporate their local conditions and use the different elements available to them, especially those that reflect the COVID-19 situation in the real world. This study provides one such model, recognizing that not all possible elements may be politically or operationally feasible given the characteristics of a particular school community.

## Preprint

This article was preprinted on medRxiv (25).

## Data availability statement

Publicly available datasets were analyzed in this study. These data can be found at: https://github.com/UCLA-PHP/school.epi.abm.

## Author contributions

DM, RN, VM, OA, NA, TK, and MI are members of a UCLA Clinical and Translational Science Institute (CTSI) science team tasked with developing a simulation model that can be used to inform scenarios under which K-6 school in-person instruction can be resumed safely during the COVID-19 pandemic. DM, RN, and VM conceptualized the original model design. DM, VM, NA, and MI performed iterative literature reviews to inform the model parametrization. DM and RN implemented and validated the model. VM and MI aligned the evolving model with pragmatic features based on school district collaborations. TK and OA advised on transmission dynamics parameterization and helped characterize the scenarios considered in the model building process. MI and TK facilitated team support from the Population Health Program at CTSI. All authors contributed to the interpretation of the data and provided substantial content to the text of the article.
